# Estimation of the burden of active and life-time epilepsy: A meta-analytic approach

**DOI:** 10.1111/j.1528-1167.2009.02481.x

**Published:** 2010-01-07

**Authors:** Anthony K Ngugi, Christian Bottomley, Immo Kleinschmidt, Josemir W Sander, Charles R Newton

**Affiliations:** * The Centre for Geographic Medicine Research – Coast (CGMR - Coast)KEMRI, Kilifi, Kenya; † Infectious Disease Epidemiology Unit, London School of Hygiene and Tropical MedicineLondon, United Kingdom; ‡ Department of Clinical and Experimental Epilepsy, UCL Institute of NeurologyQueen Square, London, United Kingdom; § SEIN – Epilepsy Institutes in the Netherlands FoundationAchterweg 5, Heemstede, The Netherlands; ¶ Clinical Research Unit, London School of Hygiene and Tropical MedicineLondon, United Kingdom; # Neurosciences Unit, Institute of Child Health, University College LondonUnited Kingdom

**Keywords:** Epilepsy, Prevalence, Burden, Meta-analysis

## Abstract

**Purpose:**

To estimate the burden of lifetime epilepsy (LTE) and active epilepsy (AE) and examine the influence of study characteristics on prevalence estimates.

**Methods:**

We searched online databases and identified articles using prespecified criteria. Random-effects meta-analyses were used to estimate the median prevalence in developed countries and in urban and rural settings in developing countries. The impact of study characteristics on prevalence estimates was determined using meta-regression models.

**Results:**

The median LTE prevalence for developed countries was 5.8 per 1,000 (5th–95th percentile range 2.7–12.4) compared to 15.4 per 1,000 (4.8–49.6) for rural and 10.3 (2.8–37.7) for urban studies in developing countries. The median prevalence of AE was 4.9 per 1,000 (2.3–10.3) for developed countries and 12.7 per 1,000 (3.5–45.5) and 5.9 (3.4–10.2) in rural and urban studies in developing countries. The estimates of burden for LTE and AE in developed countries were 6.8 million (5th–95th percentile range 3.2–14.7) and 5.7 million (2.7–12.2), respectively. In developing countries these were 45 (14–145) million LTE and 17 (10–133) million AE in rural areas and 17 (5–61) million LTE and 10 (5–17) million AE in urban areas. Studies involving all ages or only adults showed higher estimates than pediatric studies. Higher prevalence estimates were also associated with rural location and small study size.

**Conclusions:**

This study estimates the global burden of epilepsy and the proportions with AE, which may benefit from treatment. There are systematic differences in reported prevalence estimates, which are only partially explained by study characteristics.

Epilepsy is one of the most common neurologic conditions in the world, but the current estimates of 50 million people worldwide (WHO, 2004) lack precision and do not provide an estimate of the proportion with active epilepsy (AE), that is, those who may benefit from treatment.

The epidemiologic studies describing the burden of epilepsy in the last 40 years are problematic (Kotsopoulos et al., 2002; Kotsopoulos et al., 2005). Data on epilepsy are still scarce in many parts of the world, whereas the available data are inconsistent because of differences in sampling frames, case definitions, measurements (e.g., point vs. period or lifetime prevalence), screening tools, diagnostic accuracy, and different methodologic approaches (Leonardi & Ustan, 2002).

In the developed world where routine medical statistics are available and easily accessible, investigators have used research and hospital databases rather than population-based studies to estimate the prevalence of epilepsy. This practice, however, discriminates against those who underutilize medical services (Wright et al., 2000; Morgan et al., 2000). Community-based surveys are more commonly used in developing countries, but often do not make use of validated tools to screen the population. Even where validated tools are used, these studies may have higher sensitivity for convulsive epilepsies, and thus more subtle forms of epilepsy are underestimated (Da Mota et al., 2002; Racoosin, 2003).

The prevalence of epilepsy is reported to vary substantially between developed and developing countries: estimated as 4–7 per 1,000 persons in the developed countries, (Sander & Shorvon, 1996) and 5–74 per 1,000 persons in developing countries (Preux & Druet-Cabanc, 2005). The wider variations in the estimates of prevalence from resource-poor compared to developed countries complicate the use of these data in estimating the number who may benefit from treatment and in informing public health policy.

Heterogeneity in prevalence estimates, although anecdotally referred to, has not been investigated systematically. The heterogeneity could be due to differences in the prevalence of causes, case definitions, or case ascertainment. Knowledge of these factors would be useful in the design and implementation of multisite studies of epilepsy. Furthermore, differences in causes could have implications in resource allocation in public health interventions.

We conducted a systematic review of published literature to determine heterogeneity in prevalence between studies and to provide estimates of the global burden of epilepsy, in particular to provide numbers of those with AE who may benefit from treatment. Furthermore, we modeled the influence of study level covariates on the prevalence estimates.

## Methods

### Literature searches

Online databases; MEDLINE, EMBASE, PsycINFO, African Index Medicus, Index Medicus for South East Asia, Index Medicus for Eastern Mediterranean Region, BVS Virtual Health Library (Lilacs, Adolec, Medcarib, PAHO, and WHOLIS), SIGLE, Proquest, Wang Fang Database of English and Chinese online journals published in mainland China, SCIELO, CINAHL, and Global Health were systematically searched by the first author. Reference lists of identified articles were also searched for relevant titles and these were in turn searched online.

### Search strategies

Where applicable, combined text words and Medical Subject Headings (MeSH) terminology were used in addition to the two main search terms [Epilepsy & Prevalence] to identify relevant articles (Table S1). Boolean operators were used to combine search terms as necessary, and the MeSH subheadings tree was used to increase the specificity of the search terms in MEDLINE and EMBASE databases. The review question was broken down into search terms/elemental facets to develop a search strategy (Table S1). This involved the use of the recommendations of the National Health Service Centre for Reviews and Disseminations (Khan et al., 2001).

### Study selection

We included retrospective, cross-sectional, or prospective population-based studies measuring prevalence of epilepsy from anywhere in the world. Hospital-based and medical records/research database studies were also examined. The estimate of the prevalence was obtained from papers that met the criteria outlined below, which included the International League Against Epilepsy (ILAE) definition of LTE and AE (Commission on Epidemiology and Prognosis: International League Against Epilepsy, 1993). An additional definition of AE that encompasses seizures within the previous 12 months was also examined, since this is the criteria used for treatment in many developing countries.

### Inclusion and exclusion criteria

A study was included if it reported prevalence of LTE or AE; collected data using standardized previously validated questionnaires in door-to-door surveys, valid hospital and research databases, and general practice records; provided the denominator to allow recalculation of the presented or required estimates; and, included a definition of epilepsy as two or more unprovoked seizures occurring at least 24 h apart.

A study was excluded if it examined only acute symptomatic seizures, specific seizure patterns, or epileptic syndromes, for example, absence seizures; was published as a review, an editorial, an abstract only, a letter, or a comment; was a study on subpopulations, for example, prevalence of epilepsy on patients with a history of head trauma; or, was a part of duplicate populations, that is, those in which the same population overlapped different reports.

### Data extraction

We extracted data using a form designed form to capture the information of interest from the articles for this review. AKN extracted all the data, whereas CRJCN reextracted data from a sample of 10% of the studies. From each included study we obtained information on author, country, study type, study population, data collection and ascertainment method(s), age of study subjects, and whether the estimate was point or period prevalence. We used only studies that reported crude prevalence. We calculated the 95% confidence interval (95% CI) around the estimates where these were not provided. All meta-analyses were carried out in STATA 10 (StataCorp, College Station, TX, U.S.A.).

### Analysis

In the summary tables, crude prevalence estimates expressed as the number of cases per 1,000 population were presented with their 95% CIs. For all meta-analyses, models were fitted to logit-transformed observed prevalences. Estimates of the median and 5th and 95th percentiles of the distribution of true prevalences (i.e., the distribution of study prevalences that excludes variation due to sampling error) were obtained by back-transforming estimates on the logit scale to the prevalence scale.

The data were stratified on the World Bank classification of level of economic development of the study country (The World Bank, 2006), but because there were few studies, the countries were classified as developed or developing. Studies from developing countries were stratified further into urban and rural. Studies were also classified by age into those on all age groups (both children and adults), those on adults only (>15 years of age), and those on children only (≤15 years of age). Studies reporting crude LTE and AE prevalences were analyzed separately.

### Description of heterogeneity

We used forest plots (Lewis & Clarke, 2001) to visualize the heterogeneity among the studies. The standard test for heterogeneity, the Cochran chi-square (χ^2^) test, was used to examine the null hypothesis that the observed heterogeneity was due sampling error (Higgins & Thompson, 2002). Because heterogeneity was expected a priori due to clinical and methodologic diversity in the studies, we also quantified the degree of heterogeneity across studies using the statistic *I*^2^ = ((Q−df)/Q) x 100%, where *Q* is the Cochran chi-square statistic and *df* is its degrees of freedom (Higgins & Thompson, 2002; Higgins et al., 2003). *I*^2^ describes the percentage of the variability in estimates that is due to true heterogeneity (true differences in prevalence) rather than sampling error. A value >50% is considered as substantial heterogeneity.

The median of the logit-transformed prevalences was estimated from the random effects model using the command “meta” in STATA (StataCorp). In addition the 5th and 95th percentiles were estimated as *m ±* 1.96τ, where *τ* is the standard deviation of the random effect, that is, the standard deviation of the true study prevalences on the logit scale. These quantities were then back-transformed to the original prevalence scale. This approach uses information on prevalence and study size (or equivalently, standard errors/confidence intervals) and is applicable when there is significant heterogeneity (Goodman, 1989). It involves an assumption that the outcomes (such as logit prevalences) being estimated in the different studies are not identical, but follow a normal distribution, allowing for among-study variation (Goodman, 1989).

### Estimation of the number of epilepsy cases

Data on the mid-year population sizes of developed countries were obtained from the U.S. Census Bureau, International Data Base, (U.S. Census Bureau, 2007). Rural and urban population sizes in developing countries were obtained from the Columbia University’s Global Rural-Urban Mapping Project (GRUMP) database (Center for International Earth Science Information Network, 2009). The numbers of cases of LTE and AE were estimated by multiplying the estimated median prevalence obtained from the meta-analysis by the average size of the population during the period in which studies in this review were conducted. A range was obtained using the 5th and 95th percentiles.

### Investigation of the sources of heterogeneity

The following five study level covariates were investigated for their association with prevalence estimates: level of economic development, age of study participants, method of data collection, type of estimate (point or period prevalence), and study size. The influence of these variables on study prevalence was investigated using random effects meta-regression models. The models were fitted using the “metareg” command in STATA (StataCorp). This approach assumes two additive components of variance, one representing the variance within studies (i.e., error variance), and the other the variance between studies. The regression coefficients represent log odds ratios (ORs), since the models are fitted to logit-transformed data. The proportion of heterogeneity explained by each of the covariates was estimated by comparing the between-studies component of variance in the null model (τ_0_^2^) with the estimate of *τ*^2^ for the model including covariates ((τ_0_^2^–*τ*^2^)/τ_0_^2^).

Both univariate and multivariable meta-regression were performed. Variables that were significant in the univariate analysis were included in the multivariable model using a forward-selection strategy. The order in which variables were introduced into the multivariate model was determined by the size of the p-value in the univariate analysis (starting with the smallest p-value). No further variables were introduced when p > 0.05 for the introduced variable.

## Results

### Studies identified

**Figure 1:**
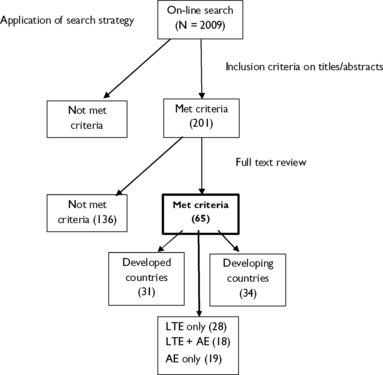
Literature search and identification of studies for the meta-analysis.

Literature searches from all sources were as displayed in Fig. 1. Reasons for exclusion of the 136 studies that underwent full text review are displayed in Table S2. Of the 65 studies included (Tables S3a and b), 28 reported LTE prevalence only, 18 reported both LTE and AE prevalence, and 19 reported prevalence of AE only. Thirty-four were from developing countries and 31 were from developed countries. Among studies from developing countries, LTE was reported in 16 studies from rural areas and 9 studies from urban areas. AE was reported in nine studies from rural areas and four from urban areas. Thirty-seven studies were conducted in both adults and children, 17 were in children only, and 11 were in adults only.

Period prevalence was estimated in 20 of the studies, whereas point prevalence was estimated in the rest. The studies did not all use the same methods for data collection: 20 studies used primarily medical records, 35 used questionnaires in cross-sectional field surveys, and 10 used medical records to ascertain cases identified through questionnaires. Sixty reports were written in English, four were Spanish, and one was in French.

Three studies from developing countries defined AE as epilepsy in which the last seizure occurred in the previous 12 months.

### Description of heterogeneity for studies of LTE

**Figure 2:**
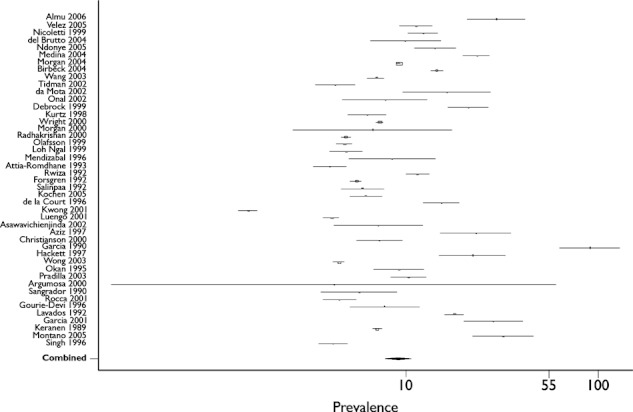
Forest plot for life-time epilepsy (LTE) prevalence per 1,000 persons (all studies).

Most of the variability in prevalence estimates was attributable to study heterogeneity (*I*^2^ = 98%, p < 0.001) (Fig. 2), both from developed (*I*^2^ >99%; p < 0.001) and developing countries (*I*^2^ = 98%; p < 0.001) (Figs. S1 and S2, respectively). The estimates also showed significant heterogeneity (I^2^ > 90%) after stratifying on age of study subjects and rural/urban locations for developing countries.

The estimated median prevalence for developed countries was 5.8 per 1,000 (5th–95th percentile range 2.7–12.4). In developing countries, the median prevalence and range of LTE was 15.4 per 1,000 (4.8–49.6) in rural areas and 10.3 per 1,000 (2.8–37.7) in urban areas (Table 1).On stratification by the age of study subjects, the median prevalence and 5th–95th percentile range was: 9.7 per 1,000 (4.6–20.5) for all age groups, 11.3 per 1,000 (4.2–30.5) for studies with adults only, and 6.7 per 1,000 (1.6–27.2) for studies of children only.

**Table 1:** Median prevalence and numbers of cases of LTE and AE

Epilepsy type	Region	Median prevalence/1,000 (5th–95th percentile range)	Mean population*a*	No. of cases in millions median (5th–95th percentile range)	Percent LTE with AE
LTE	Developed	5.8 (2.7–12.4)	1,184,235,962	6.8 (3.2–14.7)	84
	Developing	Rural*b* = 15.4 (4.8–49.6) Urban = 10.3 (2.8–37.7)	2,929,891,835 1,619,261,754	45 (14–145) 17 (10–133)	38 59
AE	Developed	4.9 (2.3–10.3)	1,184,235,962	5.7 (2.7–12.2)	
	Developing	Rural*c* = 12.7 (3.5–45.4) Urban = 5.9 (3.4–10.2)	2,929,891,835 1,619,261,754	17 (5–61) 10 (5–17)	

a Averaged over the period the selected studies were conducted.

b One study mixed rural and urban populations (not included in these analyses).

c One study mixed rural/urban populations and one unknown (both not included in the analysis).

AE, active epilepsy; LTE, life-time epilepsy.

### Description of heterogeneity for studies of AE

The estimated median prevalence of AE for developed countries was 4.9 per 1,000 (5th–95th percentile range 2.3–10.3). In the developing countries, the median prevalence and range of AE was 12.7 per 1,000 (3.5–45.4) in rural areas and 5.9 per 1,000 (3.4–10.2) in urban areas (Table 1).

When stratified on age of study participants, the median prevalence and range was 7.0 per 1,000 (2.9–16.8) for all ages, 7.0 per 1,000 (2.4–20.6) for adults, and 4.7 per 1,000 (3.3–6.9) for pediatric studies. There was substantial heterogeneity in the estimates, (*I*^2^ = 90%, p < 0.001).

### Estimates of the number of epilepsy cases

The estimated median number of people with LTE in developed countries was 6.8 million (5th–95th percentile range 3.2–14.7 million) and for AE it was 5.7 million (2.7–12.2 million). In the developing countries, the median and range of LTE cases were 45 million (14–145 million) in rural areas and 17 million (5–61 million) in urban areas. AE constituted 38% of LTE cases in rural and 59% in urban areas (Table 1).

### Sources of heterogeneity in studies of LTE prevalence

In the univariate analysis, study size explained 45.3% of the observed heterogeneity and studies with fewer than 1,000 subjects were more likely to have higher prevalence estimates than were larger studies (p < 0.001). The development level of the study country explained 26.4% of the between-study variance. In developing countries, studies from both urban and rural areas had roughly 2 times or more the prevalence of those from developed countries (Table 2). Age of subjects, method of data collection, and type of estimate were not associated with prevalence. In the multivariable regression for all LTE studies, rural areas of developing countries, studies in all age groups, and small studies (n ≤ 20,000) were significantly associated with the prevalence estimates (Table S4); together, these variables accounted for 52.8% of the observed heterogeneity.

**Table 2:** Random-effects meta-regression of prevalence of life-time epilepsy (LTE) from all studies, univariate analyses (n = 46)

Covariate	Categories (1st listed is reference)	No. studies	Odds ratio (95% CI)	p-value	Heterogeneity (τ^2^)	Heterogeneity (%)
Null model	–	46	−	−	0.53	−
Development	Developed	20	1.0	−		
	Developing (Urban)	9	1.8 (1.1– 3.0)	0.03	0.39	(26.4)
	Developing (Rural)	16	2.7 (1.8–4.0)	<0.001		
Age	Adult	7	1.0	–		
	Children	11	1.2 (0.7–2.2)	0.6	0.52	(2.0)
	All	28	0.7 (0.4–1.2)	0.2		
Data collection	Records	11	1.0	–		
	Questionnaires	29	1.6(0.8–3.0)	0.2	0.52	(1.7)
	Records and questionnaires	6	1.2 (0.6– 2.4)	0.7		
Study size	>20,000	19	1.0	−	−	−
	1,000–20,000	22	1.9 (1.4–2.7)	<0.001	0.29	(45.3)
	<1,000	5	5.2 (2.9–9.5)	<0.001		
Estimate type	Period	15	1.0	−	0.54	(−2.3)
	Point	31	1.0 (0.6–1.5)	0.9		

CI, confidence interval.

### Sources of heterogeneity in studies of AE prevalence

In the univariate analysis of all AE studies, country development level and study size were significantly associated with prevalence estimates (p < 0.05), explaining 31.7% and 26.4% of the observed heterogeneity, respectively. In the developing countries, studies from rural areas had significantly higher prevalence estimates (OR 2.5, 95% CI 1.7–3.8) relative to studies from developed countries. Small study size (n < 1,000) was also associated with higher prevalence estimates (OR 3.4, 95%CI 1.7–6.6). In the multivariable analysis, rural areas and small study size (n < 1,000) were significantly associated with prevalence estimates and together accounted for 42% of the observed heterogeneity (Table S5).

## Discussion

This study describes the distribution of prevalence in studies of people with LTE and AE across the world. Numbers of cases of LTE are provided for developed countries as well as for rural and urban locations of developing countries. Combined, these numbers provide a global estimate of cases of LTE that could be much higher than the figure of 50 million estimated by the World Health Organization (WHO) (WHO, 2004). The number of people with AE who should be considered for treatment in each region is also estimated. The studies included in these analyses, however, showed considerable heterogeneity, which we quantified using robust meta-analysis (Egger et al., 1997a, b; Higgins & Thompson, 2002; Higgins et al., 2003). There was substantial variation in the prevalence of both LTE and AE, even within studies of similar age group or level of economic development. Other estimates of the prevalence of LTE from developed countries, (Sander & Shorvon, 1996) and from developing countries, (Preux & Druet-Cabanc, 2005) are within the ranges reported in this study.

In this meta-analysis the prevalence of LTE is higher in studies of adults than studies of all ages (both adults and children), whereas it is lowest in children. The median prevalence of AE was similar for studies on all ages and adults only, but lower in studies on children. These data also showed that small studies (n < 1,000), and studies conducted in less-developed regions were associated with a higher prevalence of epilepsy. In developing countries, these data show that the prevalence of LTE is highest in rural areas, with the urban estimates being midway between those of rural areas and developed countries. In addition, the prevalence of AE in urban areas of developing countries is closer to that of developed countries, with that of rural areas being considerably higher.

To the best of our knowledge, this is the first study that comprehensively reviews and analyzes available literature to provide robust estimates of the global burden of epilepsy, assesses and quantifies the variability of the estimates, and investigates the influence of study-level covariates on the observed heterogeneity. The few reviews conducted previously have been regional, for example, Latin America (Burneo et al., 2005), exploring incidence and prevalence only (Burneo et al., 2005), incidence only (Kotsopoulos et al., 2002; Kotsopoulos et al., 2005), or mortality only (Diop et al., 2005; Forsgen et al., 2005). Furthermore, this is the first study that provides an estimate of the burden of AE that could benefit from treatment.

The difference in heterogeneity of LTE prevalence estimates between developed and resource-poor countries can be explained in part by the fact that medical records, used primarily to ascertain cases in developed countries, are to some extent standardized, and provide consistent, detailed information on patients leading to less variation in recorded data. Where available, medical records are also used to ascertain cases identified through questionnaires. Furthermore, the smaller amount of variation in studies from developed countries could be caused by the use of single district, regional, and/or national databases that use similar diagnostic codes such as the National General Practice Study of Epilepsy database in the United Kingdom. Others include use of the diagnostic record system in Rochester, an area of New York, NY, U.S.A. (Hauser et al., 1993; Da Mota et al., 2002; Racoosin, 2003; Tidman et al., 2003) or the use of the Health Maintenance Organizations’ records (Annegers et al., 1999; Holden et al., 2005).

Previously, data from developing countries were thought to vary widely due to differences in methodology (such as the use of nonstandard screening tools), and differences in definitions, diagnosis, and classification (Leonardi & Ustan, 2002; Preux & Druet-Cabanc, 2005). The selection criteria for our meta-analyses and the meta-regression models suggest, however, that these factors account for an insignificant amount of variation. Rather, age of study participants and sample size are more important causes of the observed heterogeneity. These factors may be further compounded by poor health care and lack of specialized medical personnel and diagnostic equipment. This is particularly evident given that the prevalence estimates for urban areas, with higher concentration of health facilities and specialists, are midway between those of rural areas and the developed countries. The higher estimates of LTE prevalence in developing countries are likely to be due to higher incidence of epilepsy (Sander & Shorvon, 1996), which could in turn be attributable to infectious etiology, particularly in rural areas (Ogunniyi et al., 1987; Matuja et al., 2001; Preux & Druet-Cabanc, 2005).

The trend toward a higher prevalence of AE is also apparent in rural areas of developing countries. A much lower prevalence of AE in urban areas that closely approximates estimates from developed countries could be due to better access to health services, diagnosis, and management. Rural areas of developing countries have a large burden of untreated epilepsy possibly due to stigma, beliefs and attitudes about causes and consequences of epilepsy and limited access to health services. Furthermore, recall of seizure events over a 5-year period may be poorer in rural areas due to low literacy levels and may lead to underestimation of prevalence (Saha et al., 2008).

The proportions of people with AE are higher in developed countries and urban areas of developing countries than in rural areas. This could be due to higher mortality in the latter, though few data on epilepsy mortality in developing countries are available and these are not segregated for rural and urban areas (Carpio et al., 2005; Diop et al., 2005). This could imply that people with better controlled seizures live longer on average even though they may continue to experience seizures. We have estimated the prevalence of the treatment gap to be 56% (95% CI 31–100%) in developing countries, with higher estimates for rural areas (Mbuba et al., 2008). The better access to healthcare in urban areas of developing countries and in developed countries suggests that not only management of seizures but also the less severe life-threatening etiologies improve life-expectancy.

The higher estimates of heterogeneity observed in rural areas could be due to spatial clustering of risk factors, particularly parasites (Brooker et al., 2006), associated with development of epilepsy. This observation could be partly due to clustering of genetic risk factors in rural areas, where relatives tend to live in proximity.

Small study size (fewer than 20,000 subjects screened) was associated with a higher prevalence of epilepsy, possibly because some studies are conducted in communities where the prevalence of epilepsy is suspected to be high. For instance, one study was in a small isolated population of Panamanian Indians where apparently a family history of epilepsy was a significant risk factor (risk ratio = 14) (Gracia et al., 1990).

The prevalence of epilepsy is determined by the rate at which new cases arise and the rate at which existing cases are lost due to death and recovery. The prevalence of LTE increases with age because there is, by definition, no recovery. Therefore, the older an individual is the more likely they are to have had epilepsy at some point during their lifetime. An association is observed between AE and age because of a low rate of loss of AE cases (due to recovery and death) from the population.

The variables location, age of study participants, and study size taken together account for 53% of the variance in prevalence of LTE. Therefore, much of the variation in study prevalence is attributable to factors not considered in this meta-analysis. For example, variability in the prevalence of genetic or parasitologic risk factors or the extent of the treatment gap may be responsible for some of this unexplained variation.

### Limitations of the study

The main assumption of estimates of the number of epilepsy cases is that the studies used in the analysis are representative of the populations of both developed and developing countries. However, this is hardly the case, particularly as there are no data from many parts of the world. The estimates presented in this study, therefore, need to be interpreted judiciously.

The estimates presented in these analyses are likely to be influenced by different demographic structures, particularly between developed and developing countries. However, it was not possible to derive age-adjusted estimates, mainly because studies presented different age categories, if at all.

Despite the fact that there was no time-limit criterion for inclusion, almost all the selected studies were published after 1990. This was because of the definition criteria of epilepsy used, which was introduced by the ILAE at this time (Commission on epidemiology and prognosis: International League Against Epilepsy, 1993). The definition of AE often used in less-developed regions is at least two unprovoked seizures one of which should be in the previous 12 months, but this was used in only three studies. It would have been interesting to compare the mean prevalence estimates based on this definition from a larger number of studies.

In the meta-regression analysis, the choice of covariates was influenced by the availability of information and, therefore, heterogeneity could be explained only by factors for which information was available. Ideally future studies should include more appropriate factors, for example, level of treatment gap, which may influence prevalence.

## Conclusions

This study uses a meta-analysis to provide estimates of the burden of epilepsy. We demonstrate substantial heterogeneity in estimates of the prevalence of epilepsy and identify factors responsible for this heterogeneity. This study provides estimates of the burden of AE, which can be used as a guide to the number of people who could benefit from treatment.

## Supporting Information

**Figure S1.** Forest plot for the prevalence of life-time epilepsy (LTE) per 1,000 persons (developed countries).

**Figure S2.** Forest plot for the life-time epilepsy (LTE) prevalence per 1,000 persons (developing countries).

**Table S1.** Description of search strategy.

**Table S2.** Reasons for exclusion from meta-analysis.

**Table S3a.** Summary of prevalence of epilepsy from the included studies (n = 65).

**Table S3b.** List of studies included in the meta-analysis.

**Table S4.** Random-effects meta-regression of prevalence of life-time epilepsy (LTE) from all studies, multivariable analyses (n = 46).

**Table S5.** Random-effects meta-regression of prevalence of active epilepsy (AE) from all studies, multivariable analyses (n = 37).
