# Supplementary material for: Estimation of the burden of active and life-time epilepsy: A meta-analytic approach
Source: Epilepsia. 2010 Jan 7;51(5):883–90. doi: 10.1111/j.1528-1167.2009.02481.x (PMC3410521; doi:10.1111/j.1528-1167.2009.02481.x)
Supplement: Supplementary file 1 [file epi0051-0883-SD1.doc]

**Supplementary Table 1: Description of search strategy.**

| Search Element | MEDLINE/ EMBASE/ PsycINFO | Other Databases |
| --- | --- | --- |
| *Epilepsy* **(AND)** | *Thesaurus Exploded* | *Epilepsy* (kw, ti, ab) **(AND)** |
|  | Epilepsy | *Prevalence* (kw, ti, ab) |
|  | Epilepsy, Tonic-Clonic |  |
|  | Epilepsy, Frontal Lobe |
|  | Epilepsy, Rolandic |
|  | Epilepsy, Generalized |
|  | Epilepsy, Absence |
|  | Epilepsy, Complex Partial |
|  | Myoclonic Epilepsy, Juvenile |
|  | Epilepsy, Benign Neonatal |
|  | Epilepsy, Temporal Lobe |
|  | Epilepsy, Partial, Motor |
|  | Epilepsy, Partial, Sensory |
|  | Epilepsy, Post-Traumatic |
|  | Epilepsies, Myoclonic |
|  | Epilepsies, Partial |
|  | Myoclonic Epilepsies, Progressive |
| *Epidemiology* **(OR)** | Epidemiology |
|  | +subheading “epidemiology” |
| *Morbidity* **(OR)** | Morbidity |
| *Prevalence* | Prevalence |
|  | Data collection |
|  | Cross-sectional studies |

**Supplementary Table 2: Reasons for exclusion from meta-analysis.**

| **Reason**  Lack of/inadequate definition of epilepsy (2 or more non-provoked seizures occurring at least 24 hours apart)  Study on subpopulation with epilepsy e.g. prevalence in patients with a history of head trauma  Published as a review article  Study on single epilepsy syndromes  Published as correspondence/letter/editorial/abstract only  Study on single seizure syndromes  Study of epilepsy as a risk factor to other conditions e.g. fractures  Duplicate study  Study did not provide denominator data  Study not on prevalence of epilepsy  **Total** | **No of studies**  52  6  15  23  15  5  9  4  3  4  **136** |
| --- | --- |

**Supplementary Table 3(a): Summary of prevalence of epilepsy from the included studies (n = 65).**

| **Reference** | **Country** | **Econ. Dev** | **Population** | **Ascertain*** | **Age** | **Type** | **Follow-up** | **Prevalence/1000** | **95% CI** |
| --- | --- | --- | --- | --- | --- | --- | --- | --- | --- |
| (Almu et al., 2006) | Ethiopia | Developing | 1154 | Q, E | All | LT | Point | 29.5 | 20.5 – 40.9 |
| (Velez , 2006) | Colombia | Developing | 8910 | Q, E, T | All | LT, AE | Period | 11.3 | 9.2 – 13.8 |
| (Nicoletti et al., 2005) | Bolivia | Developing | 9955 | Q, E | All | LT, AE | Point | 12.3 | 10.4 – 14.8 |
| (Del Brutto et al., 2005) | Ecuador | Developing | 2415 | Q, E | All | LT | Point | 9.94 | 5.98 – 13.9 |
| (Ndoye et al., 2005) | Senegal | Developing | 4500 | Q, E, T | All | LT | Point | 14.2 | 10.7 – 14.2 |
| (Medina, 2005) | Honduras | Developing | 6437 | Q, E, T | All | LT, AE | Point | 23.3 | 19.6 - 27 |
| (Morgan &Kerr, 2004) | UK | Developed | 424000 | MR | All | LT | Period | 9.2 | 8.9 – 9.4 |
| (Birbeck & Kalichi, 2004) | Zambia | Developing | 55000 | Q | All | AE | Point | 14.5 | 13.5 – 15.6 |
| (Wang et al., 2003) | China | Developing | 55616 | Q, E | All | LT, AE | Point | 7 | 6.3 – 7.7 |
| (Tidman et al., 2003) | UK | Developed | 15907 | MR | Children | LT | Period | 4.3 | 3.4 – 5.5 |
| (da Mota et al., 2002) | Brazil | Developing | 982 | Q, E | All | LT, AE | Point | 16.3 | 9.3 – 26.3 |
| (Onal et al., 2002) | Turkey | Developed | 2187 | Q, E, T | All | LT, AE | Period | 7.8 | 4.5 – 12.4 |
| (Debrock et al., 2000) | Benin | Developing | 3134 | Q, MR | All | LT | Point | 21.1 | 16.3 – 26.7 |
| (Kurtz et al., 1998) | UK | Developed | 17414 | Q, MR | Children | LT | Period | 6.3 | 4.9 – 7.7 |
| (Wright et al., 2000) | UK | Developed | 225439 | MR, E | All | LT, AE | Period | 7.3 | 6.9 – 7.6 |
| (Morgan et al., 2000) | UK | Developed | 434000 | MR | All | LT | Period | 6.7 | 2.0 – 13.4 |
| (Radhakrishnan et al., 2000) | India | Developing | 238102 | Q, E, T | All | LT | Point | 4.9 | 4.6 – 5.2 |
| (Olafsson & Hauser, 1999) | Iceland | Developed | 89656 | MR | All | LT | Point | 4.8 | 4.4 – 5.3 |
| (Kun et al., 1999) | Singapore | Developed | 20542 | Q, MR | Adult | LT | Point | 4.9 | 4.0 – 6.0 |
| (Mendizabal & Salguero, 1996) | Guatemala | Developing | 1882 | Q, E | All | LT, AE | Point | 8.5 | 4.9 – 13.8 |
| (Attia-Romdhane et al., 1993) | Tunisia | Developing | 25000 | Q, E | All | LT | Point | 4.04 | 3.3 – 4.9 |
| (Rwiza, 1992) | Tanzania | Developing | 18183 | Q, E | All | LT, AE | Point | 11.5 | 10.0 – 13.2 |
|  |  |  |  |  |  |  |  | Continued | |
|  | **Supplementary Table 3(a). Continued** | | | | | | | | |
| **Reference** | **Country** | **Econ. Dev** | **Population** | **Ascertain*** | **Age** | **Type** | **Follow-up** | **Prevalence/1000** | **95% CI** |
| (Forsgre, 1992) | Sweden | Developed | 129005 | MR | Adult | LT | Point | 5.53 | 5.1 – 5.9 |
| (Salinpaa, 1992) | Finland | Developed | 21104 | Q | Children | LT | Point | 5.97 | 4.0 – 6.8 |
| (Kochen & Melcon, 2005) | Argentina | Developing | 17049 | Q, E | All | LT, AE | Point | 6.2 | 5.1 – 7.5 |
| (de la Court et al., 1996) | Netherlands | Developed | 5559 | Q, MR, T | Adult | LT, AE | Point | 15.3 | 12.2 – 18.9 |
| (Kwong et al., 2001) | Hong Kong | Developed | 203499 | Q, MR, T | Children | LT | Point | 1.52 | 1.4 – 1.7 |
| (Luengo et al., 2001) | Spain | Developed | 98405 | Q, E, T | Adult | LT | Point | 4.12 | 3.7 – 4.5 |
| (Beilmann et al., 1999) | Estonia | Developed | 157449 | MR, E, T | Children | AE | Point | 3.6 | 3.3 – 3.9 |
| (Asawavichienjinda et al., 2002) | Thailand | Developing | 2069 | Q, E | All | LT | Point | 7.2 | 4.1 – 11.9 |
| (Aziz, 1997 Jun) | Pakistan | Developing | 994 | Q, E, T | Children | LT | Point | 23 | 14.7 – 34.5 |
| (Christianson et al., 2000) | S. Africa | Developed | 6692 | Q, E | Children | LT, AE | Point | 7.3 | 5.4 – 9.4 |
| (Gracia et al., 1990) | Panama | Developing | 337 | Q, E | Adult | LT, AE | Point | 90 | 60.9 – 124.6 |
| (Hackett et al., 1997) | India | Developing | 1172 | Q, E | Children | LT | Period | 22.2 | 14.5 – 32.3 |
| (Wong, 2003) | Hong Kong | Developed | 245340 | MR | Children | LT | Period | 4.5 | 4.2 – 4.8 |
| (Okan et al., 1995) | Turkey | Developed | 5002 | Q, E | Children | LT | Point | 9.2 | 6.7 – 12.2 |
| (Kraagac et al., 1999) | Turkey | Developed | 4803 | Q | All | LT | Point | 10.2 | 7.6 – 13.5 |
| (Rocca et al., 2001) | Italy | Developed | 24496 | Q, E | All | LT, AE | Point | 4.53 | 3.7 – 5.5 |
| (Gourie-Devi et al., 1996) | India | Developing | 3040 | Q | All | LT | Period | 7.8 | 5.1 – 11.7 |
| (Lavados et al., 1992) | Chile | Developing | 17694 | MR | All | LT | Point | 17.8 | 15.9 – 19.9 |
| (Argumosa & Herranz, 2000) | Spain | Developed | 225 | Q | Children | LT | Point | 4.24 | 0.12 – 24.4 |
| (Oun et al., 2002) | Estonia | Developed | 72245 | MR, E | Adult | AE | Period | 5.3 | 4.8 – 5.8 |
| (Al Rajeh et al., 2001) | S. Arabia | Developed | 22630 | Q, E, T | All | AE | Point | 6.54 | 5.5 – 7.6 |
| (Dent et al., 2005) | Tanzania | Developing | 4905 | Q, E | Children | AE | Period | 8.6 | 6.0 – 11.0 |
|  |  |  |  |  |  |  |  | Continued | |
|  |  |  |  |  |  |  |  |  | |
|  | **Supplementary Table 3(a). Continued** | | | | | | | | |
| **Reference** | **Country** | **Econ. Dev** | **Population** | **Ascertain*** | **Age** | **Type** | **Follow-up** | **Prevalence/1000** | **95% CI** |
| (Gallitto et al., 2005) | Italy | Developed | 13431 | MR, E, T | All | AE | Point | 3.13 | 2.2 – 4.2 |
| (Waaler et al., 2000) | Norway | Developed | 38593 | MR | Children | AE | Point | 5.13 | 4.4 – 5.8 |
| (Jacoby et al., 1998) | UK | Developed | 177703 | Q, MR | All | AE | Point | 7.6 | 7.2 – 7.8 |
| (Eriksson & Koivikko, 1997) | Finland | Developed | 83464 | MR | Children | AE | Point | 3.94 | 3.5 – 4.4 |
| (Aziz et al., 1997(a)) | Pakistan | Developing | 24130 | Q, MR | All | AE | Point | 9.98 | 8.8 – 11.3 |
| (Aziz et al., 1997(b)) | Turkey | Developed | 11497 | Q, MR | All | AE | Point | 7.0 | 5.5 – 8.7 |
| (Sidenvall et al., 1996) | Sweden | Developed | 36524 | Q, MR, T | Children | AE | Point | 4.2 | 3.6 – 5.0 |
| (Hauser et al., 1991) | US | Developed | 56447 | MR | All | AE | Point | 6.8 | 6.1 – 7.5 |
| (Cornaggia et al., 1990) | Italy | Developed | 54520 | Q, E, T | Adult | AE | Period | 4.7 | 4.2 – 5.3 |
| (Tran et al., 2006) | Lao PDR | Developing | 4310 | Q, E | All | AE | Point | 7.7 | 5.3 – 10.7 |
| (Fong et al., 2003) | Hong Kong | Developed | 475900 | MR, E, T | Adult | AE | Point | 1.54 | 1.4 – 1.7 |
| (Garcia-Noval et al., 2001) | Guatemala | Developing | 1183 | Q | Adult | LT,AE | Period | 28.5 | 19.3 – 39.0 |
| (Endziniene et al., 1997) | Lithunia | Developing | 88871 | MR, E, T | Children | AE | Point | 4.25 | 3.8 – 4.7 |
| (Keranen et al., 1989) | Finland | Developed | 194282 | MR, E | Adult | LT,AE | Period | 7.1 | 6.7 – 7.5 |
| (Cowan et al., 1989) | US | Developed | 246047 | MR | Adult | AE | Period | 4.71 | 4.4 – 5.0 |
| (al Rajeh et al., 1993) | S. Arabia | Developed | 22630 | Q | All | AE | Period | 6.5 | 5.5 – 7.7 |
| (Montano et al., 2005) | Peru | Developing | 903 | MR, E | All | LT,AE | Period | 32 | 22.0 – 46.0 |
| (Singh & Kaur, 1997) | India | Developing | 30000 | Q,E | All | LT | Point | 4.2 | 3.5 – 5.0 |

*Q = questionnaire; E = neurological examination; T = neuro-imaging tool (EEG, CT scan or MRI); MR = medical records; AE = active epilepsy; LT = life-time epilepsy

Only estimates of LTE are reported where the study investigated both LTE and AE.

**Supplementary Table 3(b): List of studies included in the Meta-Analysis**

Al rajeh S, Awada A, Bademosi O & Ogunniyi A. (2001 Sep) The prevalence of epilepsy and other seizure disorders in an Arab population: a community-based study. Seizure 10: 410-414.

Al rajah S, Bademosi A, Ismail H, Awada A, Awodu A, Al-freihi H, Assuhaimi S, Borollosi M & Al-shammasi S. (1993) A community survey of neurological disorders in Saudi Arabia: the Thugbah study. Neuroepidemiology 12: 164-178.

Almu S, Tadesse Z, Cooper P & Hackett R. (2006 Apr) The prevalence of epilepsy in the Zay Society, Ethiopia--an area of high prevalence. Seizure 15: 211-213.

Argumosa A & Herranz JL. (2000) The economic cost of childhood epilepsy in Spain. Rev Neurol. 30: 104-108.

Asawavichienjinda T, Sitthir-Amon C & Tanyanont W. (2002) Prevalence of epilepsy in rural Thailand: A population based study. J Med Assoc Thai 85: 1066-1073.

Attia-Romdhane N, Mrabet A & Ben Hamida M. (1993 Nov-Dec) Prevalence of epilepsy in Kelibia, Tunisia. Epilepsia 34: 1028-1032.

Aziz H, Guvener A, Akhtar SW & Hasan KZ. (1997(b)) Comparative epidemiology of epilepsy in Pakistan and Turkey: population-based studies using identical protocols. Epilepsia 36: 716-722.

Aziz H, Guvener A, Akhtar SW & Hasan KZ. ( 1997(a)) Comparative epidemiology of epilepsy in Pakistan and Turkey: population-based studies using identical protocols. Epilepsia 38: 716-722.

Beilmann A, Napa A, Soot A, Talvik I & Talvik T. (1999 Jul) Prevalence of childhood epilepsy in Estonia. Epilepsia 40: 1011-1019.

Birbeck GL & Kalichi EM. (2004 Jan) Epilepsy prevalence in rural Zambia: a door-to-door survey. Trop Med Int Health 9: 92-95.

Christianson AL, Zwane ME, Manga P, Rosen E, Venter A & Kromberg JGR. (2000) Epilepsy in rural South African children: Prevalence, associated disability and management. South African Medical Journal 90: 262-266.

Cornaggia CM, Caveninni MP, Christie W, Giuccioli D, Facheris MA, Sabbadini M & Canger R. (1990) Epidemiologic survey of epilepsy among army draftees in Lombardy, Italy. Epilepsia 31: 27-32.

Cowan LD, Bodensteiner JB, Leviton A & Doherty L. (1989) Prevalence of the epilepsies in children and adolescents. Epilepsia 30: 94-106.

De la court A, Breteler MM, Meinardi H, Hauser WA & Hofman A. (1996) Prevalence of epilepsy in the elderly: the Rotterdam Study. Epilepsia 37: 141-147.

Debrock C, Preux PM, Houinato D, Druet-Cabanac M, Kassa F, Adjien C, Avode G, Denis F, Boutros-Toni F & Dumas M. (2000 Apr) Estimation of the prevalence of epilepsy in the Benin region of Zinvie using the capture-recapture method. Int J Epidemiol. 29: 330-335.

Del Brutto OH, Santibanez R, Idrovo L, Rodriguez S, Diaz-Calderon E, Navas C, Gilman RH, Cuesta F, Mosquera A, Gonzalez AE, Tsang VC & Garcia HH. (2005 Apr) Epilepsy and neurocysticercosis in Atahualpa: a door-to-door survey in rural coastal Ecuador. Epilepsia 46: (4) 583-587.

Dent W, Helbok R, Matuja WB, Scheunemann S & Schmutzhard E. (2005 Dec) Prevalence of active epilepsy in a rural area in South Tanzania: a door-to-door survey. Epilepsia 46: 1963-1969.

Endziniene M, Pauza V & Micevisiene I. (1997) Prevalence of childhood epilepsy in Kaunas, Lithuania. Brain & Development 19: 379-387.

Eriksson KJ & Koivikko MJ. (1997) Prevalence, Classification and Severity of Epilepsy and Epileptic syndromes in children. Epilepsia 38: 1275-1282.

Fong GC, Mak W, Cheng TS, Chan KH, Fong JK. & Ho SL. (2003) A prevalence study of epilepsy in Hong Kong. Hong Kong Med J. 9: 252-257.

Forsgren L. (1992 May-Jun) Prevalence of epilepsy in adults in northern Sweden. Epilepsia., 33, 450-8.

**Supplementary Table 3(b). Continued**

Gallitto G, Serra S, la Spina P, Postorino P, Lagana A, Tripodi F, Gangemi S, Calabro S, Savica R, di Perri R, Beghi E & Musolino R. (2005 Nov) Prevalence and characteristics of epilepsy in the Aeolian islands. Epilepsia 46: 1828-1835.

Garcia-Nova J, Moreno E, de Mata F, Soto de Alfaro H, Fletes C, Craig PS & Allan JC. (2001) An epidemiological study of epilepsy and epileptic seizures in two rural Guatemalan communities. Ann Trop Med Parasitol. 95: 167-175.

Gourie-Devi M, Satishchandra G, Subbakrishna P & Subbakrishna DK. (1996) Neuro-epidemiological pilot survey of an urban population in a developing country. A study in Bangalore, south India. Neuroepidemiology 15: 313-320.

Garcia F, de Lao S & Castillo L. (1990) Epidemiology of epilepsy in Guaymi Indians of Bocas del Toro province, republic of Panama. Epilepsia 31: 718-723.

Hackett RJ, Hackett L & Bhakta P. (1997) The prevalence and associated factors of epilepsy in children in Calicut District, Kerala, India. Acta Paediatr. 86: 1257-1260.

Jacoby A, Buck D, Baker G, Mcnamee P, Graham-Jones S & Chadwick D. (1998 Jul) Uptake and costs of care for epilepsy: findings from a UK regional study. Epilepsia 39: 512-519.

Keranen T, Riekkinen PJ & Sillanpaa M. (1989) Incidence and prevalence of epilepsy in adults in eastern Finland. Epilepsia 30: 413-421.

Hauser WA, Annergers JF & Kurkland LT. (1991) Prevalence of epilepsy in Rochester, Minnesotta: 1940 - 1980. Epilepsia 32: 429-445.

Kochen S & Melcon MO. (2005 Dec) Prognosis of epilepsy in a community-based study: 8 years of follow-up in an Argentine community. Acta Neurol Scand. 112: 370-374.

Kraagac N, Yeni SN, Senocak M, Bozluolcay M, Savrun FK, Ozdemir H & Cagatay P. (1999 May) Prevalence of epilepsy in Silivri, a rural area of Turkey. Epilepsia 40: 637-642.

Kun LN, Ling LW, Wah YW & Lian TT. ( 1999 Oct) Epidemiologic study of epilepsy in young Singaporean men. Epilepsia 40: 1384-1387.

Kurtz Z, Tookey P & Ross E. (1998 Jan 31) Epilepsy in young people: 23 year follow up of the British national child development study. BMJ 316: 339-342.

Kwong KL, Chak WK, Wong SN & So KT. (2001) Epidemiology of childhood epilepsy in a cohort of 309 Chinese children. Pediatr Neurol. 24: 276-282.

Lavados JG, Morales A, Campero M & Lavados P. (1992) A descriptive study of epilepsy in the district of El Salvador, Chile, 1984 - 1988. Acta Neurol Scand. 85: 249-256.

Luengo A, Parra J, Colas J, Ramos F, Carreras T, Fernandez-Pozos MJ, Munoz A & Hernando V. (2001 Sep) Prevalence of epilepsy in northeast Madrid. J Neurol. 248: 762-767.

Medina MT, Martinez L, Osorio JR, Estrada AL, Zuniga C, Cartagena D, Collins JS, Holden KR. (2005 Jan) Prevalence, incidence, and etiology of epilepsies in rural Honduras: the Salama Study.. Epilepsia 46: 124-131.

Mendizabal JE & Salguero LF. (1996 Apr;) Prevalence of epilepsy in a rural community of Guatemala. Epilepsia 37: 373-376.

Montano SM, Villaran MV, Ylquimiche L, Figueroa LJ, Rodriguez S, Bautista CT, Gonzalez AE, Tsang VC, Gilman RH & Garcia HH. (2005) Neurocysticercosis: association between seizures, serology, and brain CT in rural Peru. Neurology 65: 229-233.

Morgan CL & Kerr MP. (2004 Jul) Estimated cost of inpatient admissions and outpatient appointments for a population with epilepsy: a record linkage study. Epilepsia 45: 849-854.

**Supplementary Table 3(b). Continued**

Ndoye NF, Sow AD, Diop AG, Sessouma B, Sene-Diouf F, Boissy L, Wone I, Toure K, Ndiaye M, Ndiaye P, de Boer H, Engel J, Mandlhate C, Meinardi H, Prilipko L & Sander JW (2005 Mar). Prevalence of epilepsy its treatment gap and knowledge, attitude and practice of its population in sub-urban Senegal an ILAE/IBE/WHO study. Seizure 14: 106-111.

Nicoletti A, Reggio A, Bartoloni A et al. Prevalence of epilepsy in rural Bolivia: a door-to-door survey. Neurology. 1999 Dec 10;53(9):2064-9.

Okan N, Okan M, Eralp O & Aytekin AH. (1995) The prevalence of neurological disorders among children in Gemlik (Turkey). Dev Med & Child Neurol. 37: 597-603.

Olafsson E & Hauser WA. (1999 Nov) Prevalence of epilepsy in rural Iceland: a population-based study. Epilepsia 40: 1529-1534.

Onal AE, Tumerdem Y, Ozturk MK, Gurses C, Baykan B, Gokyigit A & Ozel S. (2002 Sep) Epilepsy prevalence in a rural area in Istanbul. Seizure 11: 397-401.

Oun A, Haldre S & Magi M. (2002 Jan) Prevalence of adult epilepsy in Estonia. Epilepsy Res., 52, 233-42.

Radhakrishnan K, Pandian JD, Santhoshkumar T, Thomas SV, Deetha TD, Sarma PS, Jayachandran D & Mohamed E. (2000 Aug) Prevalence, knowledge, attitude, and practice of epilepsy in Kerala, South India. Epilepsia 41: 1027-1035.

Rocca WA, Savettieri G, Anderson DW, Meneghini F, Grigoletto F, Morgante L & Reggio A. (2001) Door-to-door prevalence survey of epilepsy in three Sicilian municipalities. Neuroepidemiology 20: 237-241.

Rwiza HT, Haule J, Matuja WB, Mteza I, Mbena P, Kilima PM, Mwaluko G, Mwang'ombola R, Mwaijande F. (1992 Nov-Dec) Prevalence and incidence of epilepsy in Ulanga, a rural Tanzanian district: a community-based study. Epilepsia 33: 1051-1056.

Sillanpaa M. (1992) Epilepsy in children: prevalence, disability and handicap. Epilepsia., 33, 444 - 49.

Sidenvall R, Forsgren L & Heijbel J. (1996 Jun) Prevalence and characteristics of epilepsy in children in northern Sweden. Seizure 5: 139-146.

Singh A & Kaur A. (1997) Epilepsy in rural Haryana - prevalence and treatment seeking behaviour. J Indian Med Assoc. 95(2): 37-39.

Tran DS, Odermatt P, Druet-Dabanac M, Barennes H, Strobel M. & Preux PM. (2006) Prevalence of epilepsy in a rural district of central Lao PDR. Neuroepidemiology 26: 199-206.

Velez A. (2006) Epilepsy in Colombia: epidemiologic profile and classification of epileptic seizures and syndromes. Epilepsia 47: 193-201.

Waaler PE, Blom BH, Skeidsvoll H & Mykletun A. (2000 Jul) Prevalence, classification, and severity of epilepsy in children in western Norway. Epilepsia 41: 802-810.

Wang WZ, Wu JZ, Wang DS, Dai XY, Yang B, Wang TP, Yuan CL, Scott RA, Prilipko LL, de Boer HM & Sander JW. (2003 May) The prevalence and treatment gap in epilepsy in China: an ILAE/IBE/WHO study. Neurology 60: 1544-1545.

Wong V. (2003) Study of seizure and epilepsy in Chinese children in Hong Kong: Period prevalence and patterns. Journal of Child Neurology 19: 19-25.

**Supplementary Table 4: Random effects meta-regression of prevalence of LT epilepsy from all studies, multivariable analyses (n = 46)**

| **Covariate** | **Categories** | **No. of studies** | **Odds Ratio** | **95% CI** | **P value** |
| --- | --- | --- | --- | --- | --- |
| Development | Developed  Urban (Developing)  Rural (Developing) | 20  9  16 | 1.0  1.5  2.1 | -  0.9 – 2.5  1.3 – 3.6 | -  0.08  <0.01 |
| Age | Adult  Children  All | 7  11  28 | 1.0  0.9  1.7 | -  0.5 – 1.4  1.0 – 2.7 | -  0.6  0.03 |
| Study size | >20,000  1,000-20,000  <=1,000 | 19  22  5 | 1.0  1.7  3.1 | -  1.1 – 2.4  1.7 – 5.5 | -  0.009  <0.001 |

Amount of heterogeneity explained = 52.8%

**Supplementary Table 5: Random effects meta-regression of prevalence of AE from all studies, multivariable analyses (n = 37).**

| **Covariate** | **Categories** | **No. of studies** | **Odds Ratio** | **95% CI** | **P value** |
| --- | --- | --- | --- | --- | --- |
| Development | Developed  Urban (Developing)  Rural (Developing) | 18  5  12 | 1.0  0.7  1.9 | -  0.3 – 1.4  1.2 – 3.0 | -  0.3  0.007 |
| Age | Adult  Children  All | 8  7  22 | 1.0  1.1  1.2 | -  0.7 – 1.8  0.8 – 1.8 | -  0.6  0.4 |
| Study size | >20,000  1,000-20,000  <=1,000 | 20  14  3 | 1.0  1.4  2.8 | -  0.9 – 2.1  1.4 – 5.9 | -  0.09  0.005 |

Amount of heterogeneity explained = 42.0%

**Supplementary Figure 1: Forest Plot for the LTE prevalence/1000 persons (developed countries).**

**Supplementary Figure 2: Forest Plot for the LTE prevalence/1000 persons (developing countries).**
